# Heterogeneity of *Candida* bloodstream isolates in an academic medical center and affiliated hospitals

**DOI:** 10.1128/spectrum.00464-25

**Published:** 2025-06-23

**Authors:** Nancy E. Scott, Elizabeth Wash, Christopher Zajac, Serin E. Erayil, Susan E. Kline, Anna Selmecki

**Affiliations:** 1 Bioinformatics and Computational Biology Program, University of Minnesota 5635, Minneapolis, Minnesota, USA; 2 Department of Microbiology and Immunology, University of Minnesota 5635, Minneapolis, Minnesota, USA; 3 Molecular, Cellular, Developmental Biology and Genetics Program, University of Minnesota 5635, Minneapolis, Minnesota, USA; 4 Department of Medicine, Division of Infectious Diseases and International Medicine, University of Minnesota 5635, Minneapolis, Minnesota, USA; University of Chicago, Chicago, Illinois, USA

**Keywords:** candidemia, antifungal drug resistance, emerging *Candida *species, serial isolates

## Abstract

Invasive *Candida* bloodstream infections (candidemia) are a deadly global health threat. Rare *Candida* species are becoming increasingly important causes of candidemia. Phenotypic data, including patterns of antifungal drug resistance, are limited. There is a geographic variation in the distribution of *Candida* species and frequency of antifungal drug resistance, meaning that regional data can have significant clinical value. Here, we report on the species distribution, frequency of antifungal drug resistance, and phenotypic variability of *Candida* bloodstream isolates from an academic medical center and five affiliated hospitals in the Minneapolis-Saint Paul region of Minnesota collected during an 18-month period from 2019 to 2021. We collected 288 isolates spanning 11 species from 119 patients. *C. albicans* was recovered from 45% of cases, followed by *C. glabrata* (35%), *C. parapsilosis* (7%), and *C. tropicalis* (2.5%), with 10% of cases representing additional rare species. We performed antifungal drug susceptibility by the EUCAST (European Committee on Antimicrobial Susceptibility Testing) method for three major drug classes. Concerningly, we identified fluconazole, micafungin, and multidrug resistance rates in *C. glabrata* that were ~2 times higher than that reported in other regions of the United States. Through analysis of serial isolates from individual patients, we identified clinically relevant within-patient differences of MIC values in multiple drug classes. We also report phenotypic data in rare non-albicans *Candida* species to address a significant gap in available data. Our results provide valuable clinical data relevant to antifungal stewardship efforts and highlight important areas of future research, including within-patient dynamics of infection and the mechanisms of drug resistance in rare *Candida* species.

**IMPORTANCE:**

*Candida* species are an important cause of hospital-acquired infection. The prevalence of species causing infections and the frequency of antifungal drug resistance have changed over time. Reporting the regional *Candida* species distribution and phenotypes has clinical significance. Current clinical microbiology practices may underestimate the within-host diversity of infecting strains, including important variation in drug susceptibility. Our study reports the results of an 18-month survey of *Candida* bloodstream infections in an academic medical center and associated hospitals in the Minneapolis-Saint Paul region of Minnesota. We identified antifungal drug resistance, including multidrug resistance, in multiple non-albicans species. By analyzing serial isolates from individual patients, we identified clinically relevant differences in drug susceptibility during infection. Our results contribute data to antifungal stewardship efforts and identify avenues of future research.

## INTRODUCTION

*Candida* species are frequent human commensals and also important opportunistic fungal pathogens (1–3). *Candida* infections can be superficial, such as oral candidiasis, or deeply invasive, including sites like the bloodstream (candidemia) or abdominal cavity. About 1.5 million cases of invasive candidiasis occur annually around the world (4).

Diverse *Candida* species are increasingly important causes of invasive candidiasis, and the global prevalence of *Candida albicans* has decreased to less than 50% of reported cases (5). Common non-albicans *Candida* pathogens include *C. glabrata* (*Nakaseomyces glabratus*), *C. parapsilosis*, and *C. tropicalis* (5). *C. glabrat*a is the second most common cause of invasive candidiasis in North America, Europe, and Australia, while *C. parapsilosis* is the second most common species isolated in Latin America (5–7). A growing percentage of invasive infections are caused by rare species and emerging pathogens (8, 9). *C. auris* is a recently emerged pathogen that has spread globally and can be transmitted between patients (10). *C. lusitaniae* (*Clavispora lusitaniae*) is closely related to *C. auris* and accounts for 2–3% of invasive candidiasis cases (9, 11). Important information about regional species variation can be provided by local epidemiological studies (12, 13).

Major antifungal drug classes are limited to azoles, echinocandins, and polyenes. Some *Candida* species have intrinsic resistance to specific antifungal drugs—for example, *C. krusei*’s (*Pichia kudriavzevii*) Erg11 protein has naturally reduced susceptibility to fluconazole (14, 15). *Candida* species also acquire drug resistance through a broad spectrum of mutations. The frequency of acquired antifungal drug resistance varies between species, drug class, and geographic regions (5, 7, 9). Fluconazole resistance is more frequent in non-albicans *Candida* species, including *C. glabrata* (~9%), *C. tropicalis* (~9–12%), and *C. auris* (~90%) (5, 16). Echinocandin resistance in *C. glabrata* is higher in North America (2.8%), compared to Europe (0.6%) and the Asia-Pacific region (0.4%) (5). Resistance rates can also vary by institution (e.g., echinocandin resistance rates of *C. glabrata* isolates range from 0 to 25% within different hospitals in the United States) (17). Multidrug resistance, defined as resistance to more than one class of antifungal drug, is a growing concern. *C. auris* is best known for rapid acquisition of multidrug resistance, but acquired multidrug resistance has also been reported in *C. albicans*, *C. glabrata*, *C. parapsilosis*, *C. tropicalis*, *C. krusei*, and *C. lusitaniae* (18–23). It is critical to understand the species distribution and the frequency of antifungal drug resistance at a local level to make appropriate therapeutic choices and prevent future outbreaks.

Rare *Candida* species lack sufficient clinical data to accurately define antifungal susceptibility cut-off values. Clinical breakpoints so far are limited to common pathogens, such as *C. albicans* or *C. glabrata* (24, 25). In the absence of clinical breakpoints, epidemiological cut-off values (ECOFFs) have been determined for some additional species (26). ECOFF values define the upper limit of the ‘wild-type’ distribution of minimal inhibitory concentrations (MICs) of multiple, independent groups of isolates for a given species (26, 27). Gathering more data from rare and emerging *Candida* pathogens is crucial to develop ECOFFs and clinical breakpoints, which can guide treatment decisions.

In the absence of antifungal drug resistance, some isolates demonstrate drug tolerance: persistent growth in drug concentrations above their MIC (28, 29). Tolerance may play a role in treatment failure that occurs in infections with drug-susceptible isolates (30, 31). Tolerance has been studied mostly in *C. albicans*. For fungistatic drugs, such as azoles, one measure of tolerance is supra-MIC growth (SMG) (31). SMG is calculated as the average growth across concentrations above an isolate’s MIC relative to a no-drug control (29). In *C. albicans*, SMG values > 0.3 have been associated with persistent infections and treatment failure, even in the absence of drug resistance (29). Azole tolerance levels in clinical isolates and non-albicans *Candida* species are not known and could impact patient outcomes (32, 33).

Serial clinical isolates from an individual patient can display phenotypic variation, including changes in antifungal drug resistance (34). Most previous studies of clinical *Candida* strains focused on only one isolate per patient (35–37). Few studies have analyzed serial clinical isolates, and the extent and impact of the within-host variation of *Candida* populations on clinical outcomes is poorly understood (34, 35, 38).

The aim of this study was to investigate the species distribution, frequency of antifungal drug resistance and tolerance, and phenotypic variability of *Candida* bloodstream infections through the prospective collection and analysis of residual clinical bloodstream isolates from an academic medical center and five affiliated hospitals in the Minneapolis-St. Paul (Twin Cities) metro area.

## RESULTS

### *Candida albicans* is the most common cause of candidemia in the Twin Cities area

**Fig 1:**
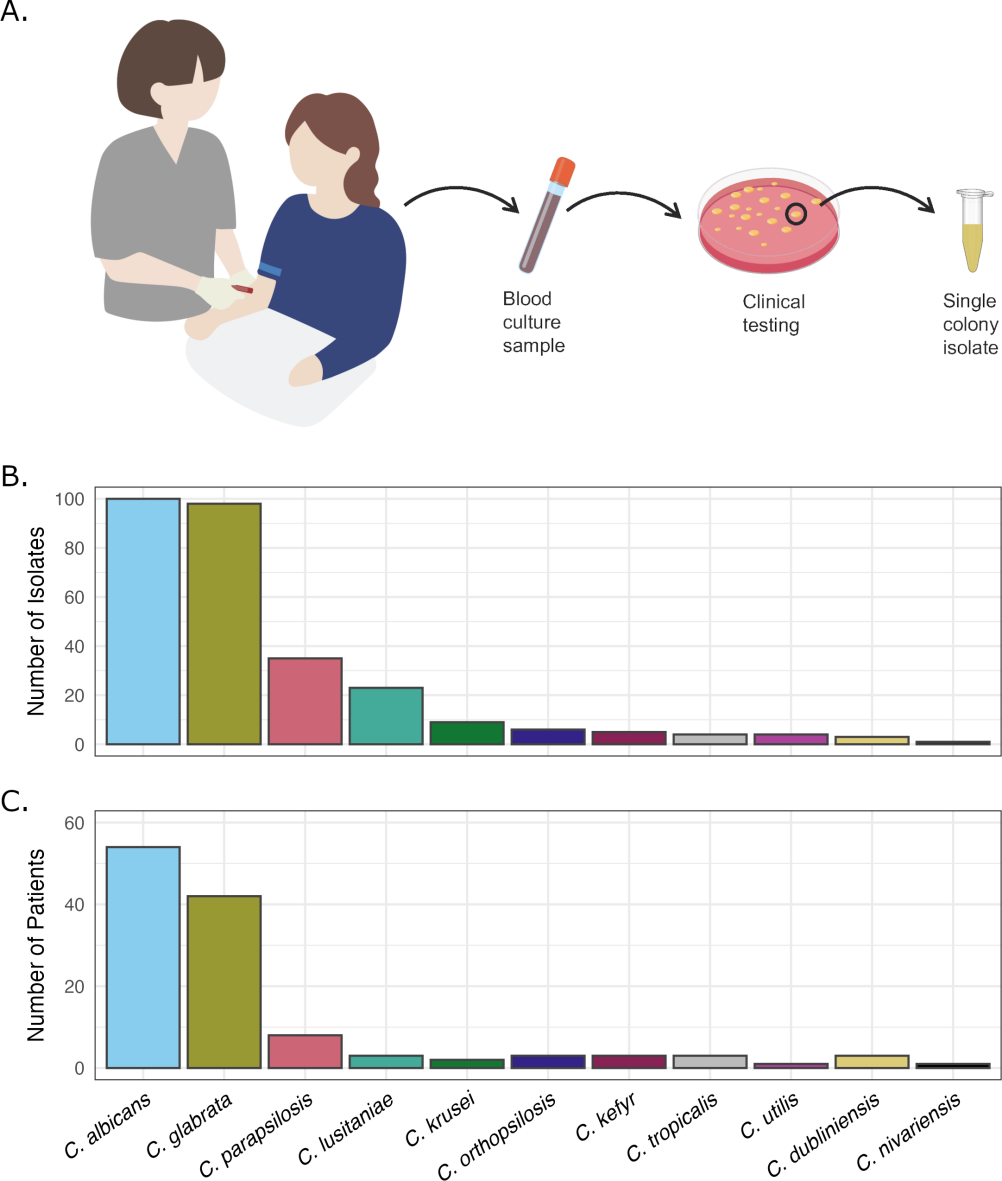
Candidemia isolate collection summary. (**A**) Workflow for isolate collection. Positive blood culture samples were subcultured for microbial identification as part of the clinical workflow. Species-level identification was performed by matrix-assisted laser desorption/ionization time-of-flight. *Candida* species were flagged by clinical staff, and a single colony was selected from the initial plate to be cultured and saved for our study. (**B**) Number of isolates collected per *Candida* species. (**C**) Number of patients with a bloodstream infection of each *Candida* species.

We collected isolates from all positive *Candida* blood cultures identified during clinical testing between December 2019 and May 2021 (see Methods and Table S1). For this study, we define an isolate as a single colony subculture taken from an individual blood culture sample (Fig. 1A). We collected a total of 288 isolates representing 11 species from 119 different patients (Fig. 1B and C). Of the four most common pathogenic *Candida* species, *C. albicans* was most frequently identified in the study and isolated from 54 patients (45.3%), followed by *C. glabrata* (*n* = 42, 35.3%), *C. parapsilosis* (*n* = 8, 6.7%), and *C. tropicalis* (*n* = 3, 2.5%). Other rare species detected in this study included *C. dubliniensis*, *C. kefyr* (*Kluyveromyces marxianus*), *C. orthopsilosis*, and *C. lusitaniae* (each isolated from three patients), *C. krusei* (two patients), *C. nivariensis* (*Nakaseomyces nivariensis*, one patient), and *C. utilis* (*Cyberlindnera jadinii*, one patient).

### Multiple isolates from individual patients demonstrate the within-host diversity of clinical strains

**Fig 2:**
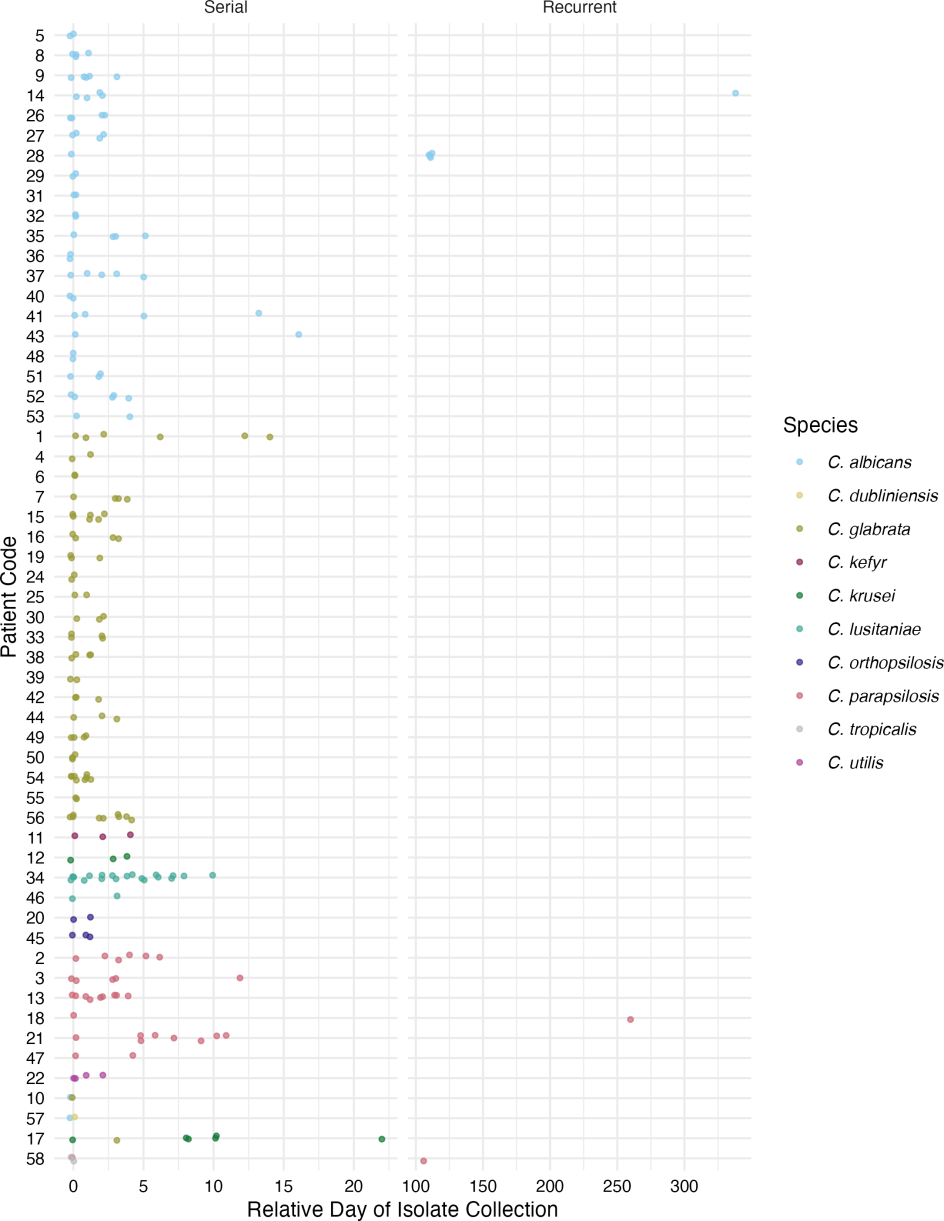
Timelines of the serial, recurrent, and polyfungal isolate sampling. Patient codes are on the *y*-axis, and the relative number of days is on the *x*-axis. Serial cases have multiple isolates of a single species collected from one patient within 30 days of the initial positive culture. Recurrent cases have additional isolates of the same species collected more than a month after the initial positive blood culture.

Forty-eight percent of patients had multiple positive blood cultures during the study period, and we collected one isolate from each positive culture. We defined a case as all isolates collected from an individual patient, and each case was assigned a numeric code that was unrelated to any patient identifiers. To provide more detailed information about isolates collected throughout individual patient infections, we further defined four categories of cases: (i) individual cases (one isolate collected from one patient); (ii) serial isolate cases (multiple isolates of a single species collected from one patient within 30 days of the initial positive culture); (iii) recurrent cases (multiple isolates of a single species collected from one patient more than 30 days after the initial positive culture); and (iv) polyfungal cases (multiple species collected from a single patient within 30 days of each other). Serial isolate cases were collected from all species, except *C. dubliniensis* and *C. nivariensis* (Fig. 2; Table S2). Four recurrent cases were identified (3.4% of all cases): two were recurrent *C. albicans* infections, and two were recurrent *C. parapsilosis* infections. The time span between the sampling of recurrent isolates ranged from 107 to 338 days (Fig. 2; Table S3).

Four polyfungal cases were collected during the study (Fig. 2; Table S3), including two cases that each involved two different species collected independently on one day (*C. albicans* and *C. glabrata* isolated from patient 10; *C. albicans* and *C. dubliniensis* isolated from patient 57). We found no patterns related to the species, which were isolated together or the time spans involved in polyfungal cases. Two patients fit into multiple categories, highlighting the complexity of some candidemia infections. Patient 17 had six *C. krusei* blood cultures collected over 22 days, with an additional single *C. glabrata* blood culture on the third day, comprising both serial and polyfungal cases. Patient 58 had two independent *C. tropicalis* blood cultures and a *C. parapsilosis* blood culture collected over the course of 2 days, and another *C. parapsilosis* blood culture over 3 months later, therefore fitting the categories of serial, polyfungal, and recurrent cases.

### Antifungal resistance is most common in *C. glabrata* but also occurs in other non-albicans species

We performed antifungal susceptibility testing on all isolates to determine the frequency of resistance against the three major antifungal drug classes. We selected fluconazole, micafungin, and amphotericin B because they are commonly used representatives of their respective drug classes (39). We measured the MIC using the European Committee on Antimicrobial Susceptibility Testing (EUCAST) broth microdilution method and interpreted results according to EUCAST breakpoints (Table S1) (24, 25). All tests were performed in triplicate. For eight of 288 isolates that had insufficient growth for EUCAST growth criteria, we measured the MIC by gradient diffusion (Methods). For species without clinical resistance breakpoints, we evaluated available ECOFF values.

Fluconazole clinical resistance breakpoints were available for all species in this study, except for *C. krusei*, which has intrinsic resistance. Twenty-three *C. glabrata* isolates (23.5%), one *C*. *tropicalis* isolate (25%), and four *C*. *utilis* isolates (100%) had fluconazole resistance (Table 1; Table S1). The clinical breakpoint of fluconazole for *C. glabrata* is 16 mg/L, and the MIC values for resistant *C. glabrata* isolates ranged from 32 to 256 mg/L. All *C. albicans*, *C. parapsilosis*, *C. lusitaniae*, *C. orthopsilosis*, *C. kefyr*, *C. dubliniensis*, and *C. nivariensis* isolates were susceptible to fluconazole.

**TABLE 1:** Antifungal susceptibility test results of *Candida* bloodstream isolates^*a*^

Species, isolates (n), antifungal	MIC range (mg/L)	MIC_50_ (mg/L)	MIC_90_ (mg/L)	EUCAST		ECOFF		EUCAST breakpoints S, R (mg/L)	ECOFF values (mg/L)
				S (%)	R (%)	WT (%)	NWT (%)		
*C. albicans, n* = 100									
Amphotericin B	0.032–1	0.5	1	100	0	100	0	≤1, >1	1
Fluconazole	0.5–1	0.5	0.5	100	0	100	0	≤2, >4	0.5
Micafungin	0.016	0.016	0.016	100	0	100	0	≤0.03, >0.03	0.03
*C. glabrata, n* = 98									
Amphotericin B	0.064–1	0.256	0.5	100	0	100	0	≤1, >1	1
Fluconazole*	0.5–256	4	128	0	23.5	76.5	23.5	*, >16	16
Micafungin	0.016–>1	0.016	0.064	89	11	88.2	11.2	≤0.03, >0.03	0.03
*C. parapsilosis, n* = 35									
Amphotericin B	0.016–1	0.5	1	100	0	100	0	≤1, >1	1
Fluconazole	0.5–2	0.5	1	100	0	100	0	≤2, >4	2
Micafungin	1–2	1	2	100	0	100	0	≤4, >4	4
*C. lusitaniae, n* = 23									
Amphotericin B	0.125–1	0.125	0.5	—	—	95.7	4.3	—	0.5
Fluconazole	0.5	0.5	0.5	100	0	100	0	≤2, >4 #	2
Micafungin	0.032–>1	0.064	0.5	—	—	69.6	30.4	—	0.125
*C. krusei, n* = 9									
Amphotericin B	1	1	1	100	0	100	0	≤1, >1	1
Fluconazole**	>32	>32	>32	NR	NR	NR	NR	NR	NR
Micafungin	0.125	0.125	0.125	—	—	100	0	—	0.25
*C. orthopsilosis, n* = 6									
Amphotericin B	0.25–0.5	0.25	0.5	—	—	—	—	—	—
Fluconazole	0.5	0.5	0.5	100	0	—	—	≤2, >4 #	—
Micafungin	0.5	0.5	0.5	—	—	—	—	—	—
*C. kefyr, n* = 5									
Amphotericin B	0.5–1	0.5	1	—	—	100	0	—	1
Fluconazole	0.5	0.5	0.5	100	0	100	0	≤2, >4 #	1
Micafungin	0.032–0.064	0.064	0.064	—	—	100	0	—	0.125
*C. tropicalis*, *n* = 4									
Amphotericin B	0.5	0.5	0.5	100	0	100	0	≤1, >1	1
Fluconazole	0.5–64	0.5	64	75	25	75	25	≤2, >4	1
Micafungin	0.016–0.032	0.016	0.032	100	0	100	0	≤0.06, >0.06	0.06
*C. utilis, n* = 4									
Amphotericin B	0.25–0.5	0.25	0.5	—	—	—	—	—	—
Fluconazole	64–160	128	128	0	100	0	100	≤2, >4 #	—
Micafungin	0.032	0.032	0.032	—	—	—	—	—	—
*C. dubliniensis, n* = 3									
Amphotericin B	0.064–0.25	0.064	0.5	—	—	100	0	—	0.25
Fluconazole	0.5	0.5	0.5	100	0	100	0	≤2, >4	0.5
Micafungin	0.032	0.032	0.032	—	—	100	0	—	0.06
*C. nivariensis, n* = 1									
Amphotericin B	1	N/A	N/A	—	—	—	—	—	—
Fluconazole	4	N/A	N/A	—	—	—	—	—	—
Micafungin	0.016	N/A	N/A	—	—	—	—	—	—

^*a*^ Abbreviations: EUCAST, European Committee on Antimicrobial Susceptibility Testing; S, susceptible; R, resistant; ECOFF, epidemiologic cut-off values; WT, wild-type; NWT, non-wild-type values; NR, not recommended; N/A not applicable. MIC_50_ is the concentration at which 50% of tested isolates were inhibited, and MIC_90_ is the concentration that inhibited 90% of the tested isolates. The asterisk (*) at *C. glabrata* indicates that isolates with fluconazole MIC ≤ 16 mg/L are susceptible, increased dose-dependent rather than susceptible. The double asterisk (**) at *C. krusei* indicates intrinsic fluconazole resistance in this species. —, EUCAST breakpoints or ECOFFs are not established and not reported. #, determined on the basis of PK/PD data, independent of specific species.

Micafungin clinical resistance breakpoints are only established for *C. albicans*, *C. glabrata*, and *C. parapsilosis*. ECOFF values are available for *C. dubliniensis*, *C. kefyr*, *C. krusei*, *C. lusitaniae*, and *C. tropicalis*. Eleven *C. glabrata* isolates (11.2%) were resistant to micafungin (Table 1; Table S1). Strikingly, seven of 23 (30.4%) *C*. *lusitaniae* isolates had micafungin MIC values greater than the established ECOFF of 0.125 mg/L (Table 1; Table S1). Notably, all micafungin-resistant phenotypes were found within a single serial case of *C. lusitaniae*, with seven of 20 serial isolates from case 34 having micafungin MIC values ranging from 0.256 to >1 mg/L micafungin (40). All *C. albicans* and *C. parapsilosis* isolates were micafungin-sensitive (Table 1; Table S1). All *C. dubliniensis*, *C. kefyr*, *C. krusei*, and *C. tropicalis* isolates had micafungin MICs below their established ECOFF values (Table 1; Table S1). No ECOFF values are available for *C. orthopsilosis*, *C. nivariensis*, and *C. utilis*. All *C. orthopsilosis* isolates had micafungin MIC values of 0.5 mg/L, which is less than the median MIC values from other studies using EUCAST broth microdilution (41, 42). The micafungin MIC values for *C. nivariensis* and *C. utilis* in this study ranged from 0.016 to 0.064 mg/L and are consistent with the median MIC values reported by other studies using EUCAST broth microdilution (42, 43).

Amphotericin B clinical resistance breakpoints are available for *C. albicans*, *C. glabrata*, *C. parapsilosis*, *C. krusei*, *C. tropicalis*, or *C. dubliniensis*. There was no amphotericin B resistance in these six species (Table 1; Table S1). A single *C. lusitaniae* isolate had an amphotericin B MIC of 1 mg/L (ECOFF value 0.5 mg/L). EUCAST now recommends that all *C. lusitaniae* isolates be reported as amphotericin B-resistant, regardless of MIC (44). All *C. kefyr* isolates had amphotericin B MIC values below the ECOFF value of 1 mg/L. All *C. nivariensis*, *C. orthopsilosis*, and *C. utilis* isolates tested for this study had MIC values of 1 mg/L or less for amphotericin B.

### *C. glabrata* micafungin and fluconazole resistance are more frequent in our study compared to that reported from other regions of the United States

To examine the frequency of resistance at the case level, we determined the number of patients with any resistant isolates (i.e., serial resistant isolates from an individual patient count as a single case). The number and percentage of resistant cases per species are summarized in Table 2. Species with no resistant isolates are not listed.

**TABLE 2:** Frequency of antifungal drug-resistant cases by species

Species	Total cases, *N*	Drug	Resistant cases, *N* (%)
*C. glabrata*	42	Fluconazole	7 (16.7)
		Micafungin	3 (7.1)
*C. lusitaniae*	3	Micafungin	2 (66.7)
		Amphotericin B	1 (33.3)
*C. tropicalis*	3	Fluconazole	1 (33.3)
*C. utilis*	1	Fluconazole	1 (100)

We found micafungin resistance in 7.1% of *C. glabrata* cases, about twice the frequency of cases resistant to any echinocandin (3.6%) reported by the CDC EIP for 2016 (9). We also identified fluconazole resistance in 16.7% of *C. glabrata* cases in our study, which is notably higher than the 10.7% reported by the CDC EIP for 2016 (9). We identified micafungin resistance in one of only three *C*. *lusitaniae* cases collected in our study. Overall, our results indicate that in *C. glabrata*, antifungal drug resistance to two major drug classes is concerningly high in the Twin Cities metro area.

### Multidrug resistance is found in non-albicans *Candida* species

Multidrug resistance is an important clinical concern because antifungal treatment options are limited. We identified multidrug resistance in 33% of *C. lusitaniae* cases (micafungin and amphotericin B, *n* = 1 patient) and in 4.8% of *C. glabrata* cases (fluconazole and micafungin, *n* = 2 patients). The frequency of multidrug resistance in our study is almost two times the national average reported by the CDC EIP in 2016 (0–2.7%) (9).

### Differences in MIC values between serial isolates occur in all three antifungal drugs

**Fig 3:**
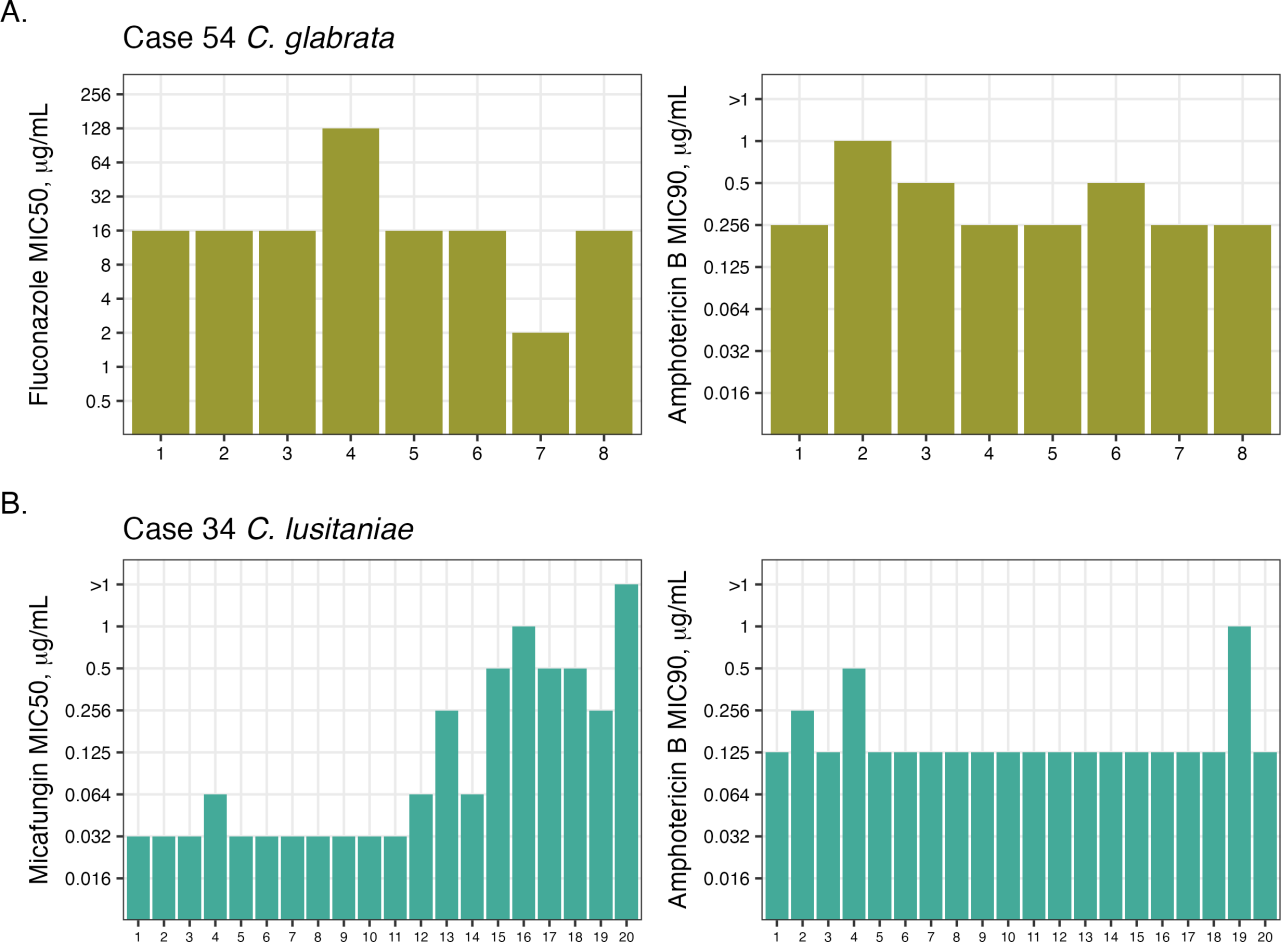
Within-host variation of MIC values occurs in all three drugs. (**A**) Sixty-four-fold fluconazole MIC differences and fourfold amphotericin B MIC differences in case 54 *C*. *glabrata* isolates. (**B**) Sixty-four-fold micafungin MIC differences and eightfold amphotericin B in case 34 *C*. *lusitaniae* isolates.

Clinical antifungal susceptibility testing is often only performed on the first isolate collected from a patient, limiting our understanding of both the within-host variation and the speed at which drug resistance is acquired during treatment. To determine how often MIC values differ between related isolates, we compared results of MIC testing performed in triplicate for all serial and recurrent cases from individual patients. Replicate MIC values for individual isolates were consistent, with up to only a twofold difference between results (a single doubling dilution). Therefore, we set a threshold of a minimum fourfold difference to be considered as differences in MIC in related isolates. Nine serial isolate cases had a four- to 64-fold variation in MIC (Table S4). Notably, two of these cases had MIC differences to multiple drugs. Patient 54, a serial case of 8 *C*. *glabrata* isolates, had a fourfold increase in amphotericin B MIC and a 64-fold increase in fluconazole MIC across the isolates, indicating a substantial within-host variation of resistance (Fig. 3A). Case 34, a *C. lusitaniae* serial case of 20 isolates, had an eightfold increase in amphotericin B MIC and a 64-fold increase in micafungin MIC (Fig. 3B). We also identified a fourfold increase in amphotericin B MICs in two *C. albicans* cases and a four- to eightfold increase in fluconazole MIC in three *C. glabrata* cases, one *C. utilis* case, and one *C. parapsilosis* case. Differences in MIC values in serial isolates might represent existing within-host diversity of a strain or might be changes that are actively being selected for during antifungal therapy.

### Fluconazole tolerance is greatest in *C. glabrata*

**Fig 4:**
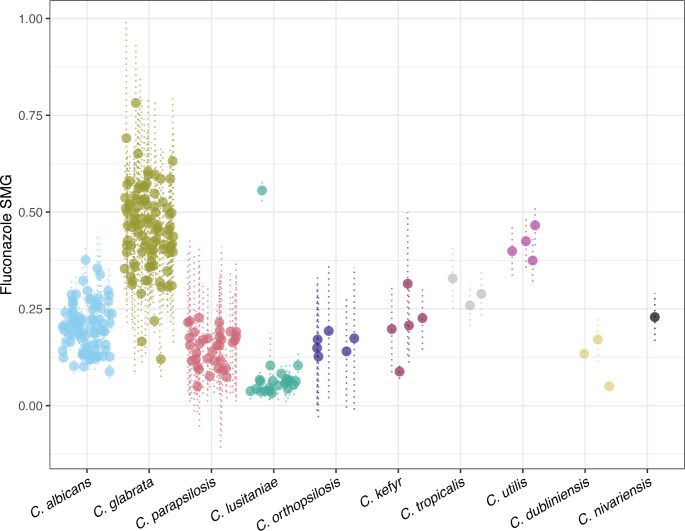
Fluconazole tolerance varies across and within *Candida* species. Dot plot of supra-MIC growth (SMG). For each isolate, the mean SMG is represented as a point, and the standard deviation is shown as dotted lines. SMG is the proportion of growth at 48 h in all drug concentrations above the MIC, relative to a no-drug control. SMG testing was performed in triplicate for all isolates.

To evaluate fluconazole tolerance (slow growth in drug concentrations above an isolate’s MIC), we determined 48 h SMG values (Fig. 4). SMG is calculated as the average growth of an isolate across drug concentrations greater than its MIC, relative to a no-drug control. *C. albicans* isolates with SMG values > 0.3 have been associated with persistent infections (29). The *C. albicans* isolates in our study had a mean SMG of 0.2, with a range of 0.09 to 0.38. All isolates belonging to recurrent *C. albicans* cases had SMG values less than 0.2, suggesting that tolerance did not play a role in recurrence.

*C. glabrata* isolates had the highest fluconazole tolerance of all species in our study, with a mean SMG of 0.497 and a range of 0.16 to 0.78 (Table S5). Despite the high SMG values, there were no recurrent *C. glabrata* cases in our study, and 19 of 20 serial isolate cases had time spans of less than 5 days. The *C. glabrata* isolates had high levels of fluconazole resistance along with tolerance; however, their MIC and SMG values were not correlated (Spearman’s rank correlation coefficient = 0.12, *P* = 0.309 after ), indicating that these are independent mechanisms of growth in the presence of drug.

*C. parapsilosis* isolates had generally low SMG values, with a mean of 0.15 and range from 0.05 to 0.23, which may indicate low tolerance but could also reflect overall slower growth in this species. Among the rare species, *C. lusitaniae* had the lowest fluconazole tolerance overall with a mean SMG of 0.08, but a single isolate had an SMG of 0.56, exceeding the tolerance of all species other than *C. glabrata*. Notably, this fluconazole-tolerant *C. lusitaniae* isolate was also resistant to micafungin and amphotericin B. When comparing SMG values of isolates within serial cases, we identified multiple instances of SMG differences ≥ 0.1 involving *C. glabrata*, *C. albicans*, *C. parapsilosis*, *C. lusitaniae*, and *C. kefyr* (Table S6). These within-patient differences in tolerance may reflect existing phenotypic variation in a strain or may be evidence of within-host evolution during treatment. Our data again highlight the value of testing multiple isolates from a patient over several days during antifungal treatment.

### There is limited association between growth rates in the absence of drug and MIC values

**Fig 5:**
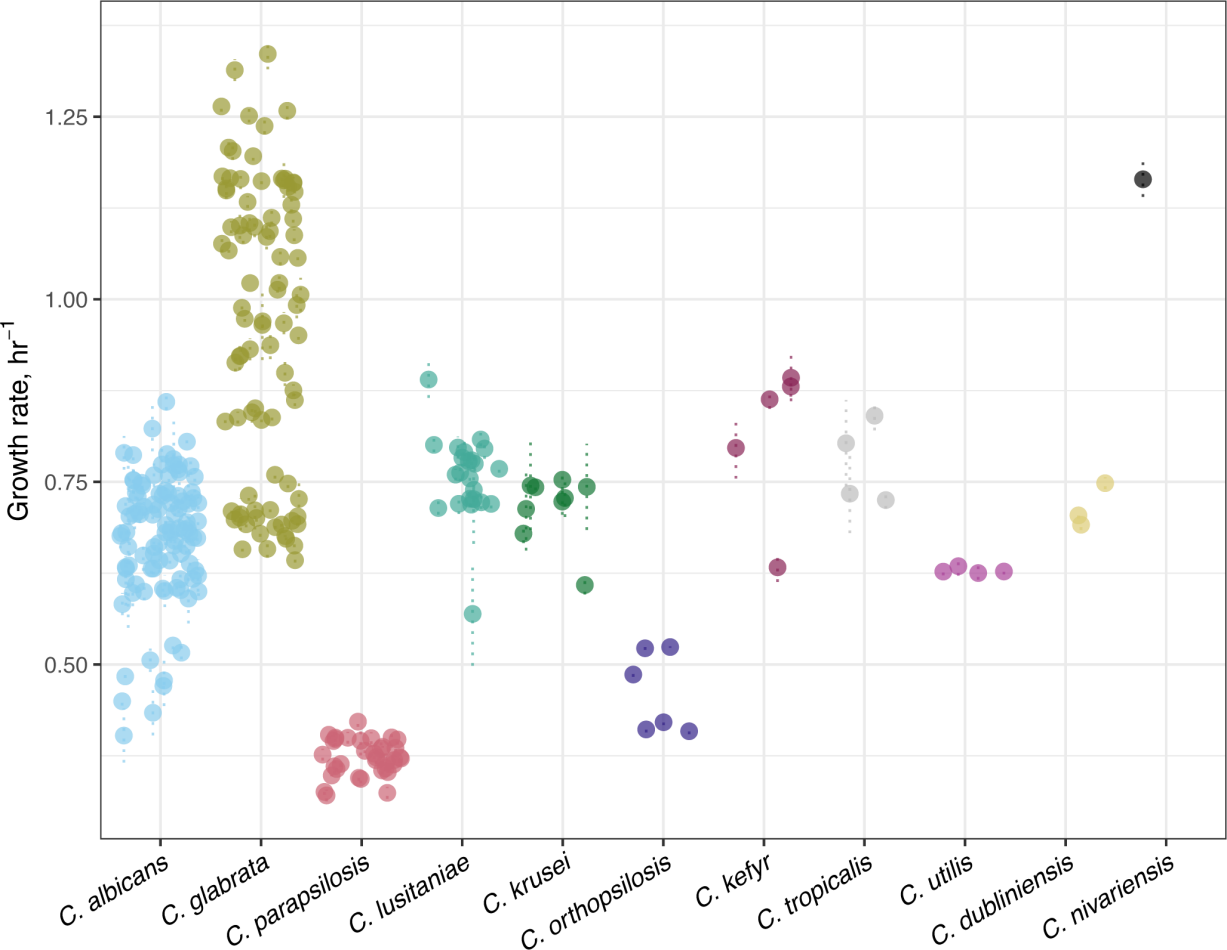
Distribution of isolate growth rate/h^−1^ in the absence of drug. For each isolate, the mean growth rate in YPAD is represented as a point, and the standard deviation is shown as dotted lines. Growth curves were performed in triplicate for all isolates.

Bacteria often have a fitness cost associated with antimicrobial resistance, but in fungi, the relationship between fitness and antifungal drug resistance is not straightforward (45). We calculated the growth rate (*r*) of all isolates as a proxy for fitness over 24 h in the absence of drug (Fig. 5). *C. glabrata* and *C. nivariensis* had the fastest overall growth rates (*C. glabrata* median *r* = 0.988, *C. nivariensis r* = 1.16). *C. parapsilosis* had the slowest growth of any *Candida* species (median *r* = 0.373).

To determine if increased MIC values are associated with a growth defect, we tested whether there was a correlation between the MIC (in each drug class) and mean growth rate (in the absence of drug) for individual species. Species with a single MIC value for a given drug were excluded. In fluconazole, we identified a significant negative correlation between growth rate and MIC in *C. glabrata* (Spearman’s rank correlation coefficient = −0.24, *P* = 0.024) and in *C. utilis* (Spearman’s rank correlation coefficient = −1, *P* = 0) but no correlation in *C. albicans*, *C. parapsilosis*, and *C. tropicalis* (Fig. S1 and Table S7). In micafungin, there was no significant correlation between growth rate and MIC for all tested species (Fig. S2 and Table S7). In amphotericin B, we identified a significant negative correlation between growth rate and MIC value in *C. orthopsilosis* (Spearman’s rank correlation coefficient = −0.87, *P* = 0.021). Surprisingly, in amphotericin B, we identified a significant positive correlation between growth rate in the absence of drug and MIC for *C. glabrata* (Spearman’s rank correlation coefficient = 0.22, *P* = 0.039) and *C. parapsilosis* (Spearman’s rank correlation coefficient = 0.49, *P* = 0.003) (Fig. S3 and Table S7). There was no correlation between MIC values and growth rates in *C. albicans*, *C. lusitaniae*, *C. kefyr*, *C. utilis*, or *C. dubliniensis*.

## DISCUSSION

Candidemia is an important hospital-associated infection with significant associated mortality. Population-based surveillance studies have revealed important geographic and temporal variations in causal species and frequencies of antifungal drug resistance. We report the species distribution and antifungal resistance for an academic medical center and five affiliated hospitals in the Twin Cities metro area of Minnesota. We prospectively collected 288 bloodstream isolates representing 11 *Candida* species from 119 patients, including 57 serial isolate cases representing nine species.

*C. albicans*, *C. glabrata*, and *C. parapsilosis* were the most frequently recovered species in our study. Our data are relatively consistent with nationwide studies from 2012 to 2017, albeit with slightly lower frequencies of C. *parapsilosis* and *C. tropicalis* (9, 46). Approximately 10% of our cases were caused by rare *Candida* species, highlighting their growing clinical importance. The frequency of polyfungal infections in our study (3.4%) is consistent with results reported by other studies in the United States and elsewhere (9, 47). The frequency of recurrent infection in this study, 3.4%, is somewhat lower than the 6% recurrence reported by a CDC EIP candidemia study of Georgia, Maryland, Oregon, and Tennessee, which could reflect regional and temporal variations (48).

We determined the frequency of antifungal drug resistance for all isolates to three drug classes. We identified no drug resistance in *C. albicans*, which is consistent with very low levels reported by the CDC EIP at four national sites (9). We also identified no resistance to any drug classes in *C. parapsilosis*. Other U.S. studies have reported higher rates of fluconazole resistance in *C. parapsilosis* bloodstream isolates (9, 49). Local fluconazole resistance might be lower due to differences in treatment practices or might reflect undersampling due to the limited number of *C. parapsilosis* cases in this study (50). We identified no amphotericin B resistance in non-*lusitaniae* isolates, which is consistent with low levels of amphotericin B resistance reported across most *Candida* species (51, 52). Notably, we identified a range of amphotericin B MIC values within a serial *C. lusitaniae* case. Within this individual patient, only one of 20 isolates had a non-wild-type MIC value. This isolate was collected within 6 days of initiation of therapy, highlighting how rapidly *C. lusitaniae* can acquire resistance to amphotericin B and why EUCAST guidance is that all isolates be considered resistant (44, 53, 54).

Importantly, in *C. glabrata*, we identified fluconazole resistance in 16.7% of cases, micafungin resistance in 7.1% of cases, and multidrug resistance in 4.8% of cases—frequencies that are 1.5–2 times higher than earlier studies of *C. glabrata* in the United States (9, 13). These results are clinically important and concerning because echinocandins are recommended first-line therapy for candidemia, and fluconazole is an important step-down treatment for candidemia (55). Our results may represent geographic variation or could reflect changes in treatment practices—in 2016, treatment guidelines for candidemia were revised to recommend echinocandins as first-line therapy in contrast to the 2009 guidelines, which indicated fluconazole was acceptable first-line treatment (55, 56). We identified multidrug resistance in a single case of *C. lusitaniae* and fluconazole resistance in one *C. utilis* case. Our results are an important addition to the very limited data available for both species. Overall, our findings emphasize that continued region-specific monitoring of antifungal drug resistance is crucial for identifying trends in resistance patterns that could impact antifungal stewardship efforts.

A strength of our study is the collection and testing of serial isolates from individual patients. We identified fourfold and greater MIC differences in nine of the 56 patients that had multiple isolates collected. In some cases, isolates collected on the same day had differing MIC values, while in other cases, MIC values climbed later during the infection or even dropped relative to earlier isolates. Our data highlight the potential clinical significance of within-host diversity and the limitations of current clinical testing strategies.

Tolerance has been associated with persistent or recurrent infection and is understudied in all *Candida* species (28). We measured SMG as an indicator of fluconazole tolerance for all isolates and have reported some of the first SMG values for non-albicans species. While other studies have identified increased SMG values in persistent *C. albicans* infections, we did not find an association with SMG and recurrence (29). However, our study collected a limited number of recurrent infections. Further investigation of tolerance mechanisms, including further longitudinal sampling and testing of additional drugs, is an important direction for future work.

We determined the growth rate in rich media for all species as a measure of fitness in the absence of drug. There was extensive variation between isolates in *C. albicans* and *C. glabrata*, the two most common species in our study. Among the species that had a range of MIC values, we identified both positive and negative associations between increases in MIC and potential growth defects in the absence of drug. Overall, our data indicate that the association between MIC and growth defects varies by species and drug class. These results suggest that there is not always a fitness trade-off associated with drug resistance in *Candida* species and highlight the need for further investigation to elucidate the relationship between acquired resistance and fitness in clinically relevant environments.

There are several limitations to our study. While serial isolates are well represented in our data set, all isolates are single colony subcultures. Our sampling strategy, while reflective of modern clinical microbiology practices, may underestimate the diversity of bloodstream populations during infection. Unstable genomic alterations, such as aneuploidy, which can be important drivers of drug resistance and tolerance (33, 57–59), may be missed as a result of this single colony subculturing.

In conclusion, our survey of species distribution of *Candida* bloodstream isolates in an academic health center and five affiliated hospitals was generally consistent with national and international trends, including the rise of rare species. While antifungal drug resistance in *C. albicans* and *C. parapsilosis* was absent, we identified important and deeply concerning trends in antifungal drug resistance in *C. glabrata* compared to other regions within the United States. The frequency of drug resistance in *C. glabrata* has implications for local antifungal stewardship efforts. We have provided valuable phenotypic data for rare *Candida* species, particularly as it relates to the understudied phenomenon of drug tolerance. We have also described within-host phenotypic variability among common and rare *Candida* pathogens, which has potential clinical significance and is an important avenue for future research.

## MATERIALS AND METHODS

### Isolate and data collection

All available *Candida* bloodstream isolates identified from patients in M Health Fairview System hospitals were collected between December 2019 and May 2021. The M Health Fairview Infectious Diseases Diagnostic Laboratory performed species-level identification of all isolates by matrix-assisted laser desorption/ionization time-of-flight. Each isolate is a single colony subculture of an individual clinical blood culture. Isolates and patients were assigned unique study codes unrelated to identifying information. Colonies were cultured on Sabouraud dextrose agar plates, and stocks were prepared with 20% glycerol and stored at −80°C.

### Minimum inhibitory concentration by broth microdilution

The MIC was determined by broth microdilution performed in RPMI 1640 (Cytiva, product no. SH30011.02) with 0.2% dextrose buffered with 0.165 M MOPS (Thermo Fisher Scientific, product no. J19256A1) and adjusted to pH 7.0 (27). Cultures were grown from glycerol stocks on Sabouraud dextrose agar plates at 35°C for 48 h. Inoculum was prepared by suspending multiple colonies into sterile water and diluted to a final OD600 of 0.01. Then, 100 µL of the inoculum was added to 96-well plates containing 100 µL of twofold serial dilution of antifungal drug in 2× RPMI medium (fluconazole: Alfa Aesar product no. J62015, micafungin: MedChemExpress product no. HY-17579, amphotericin B: Chem-Impex International product no. 00329). Plates were incubated in a humidified chamber at 35°C without shaking. At 24 h post-inoculation, plate cultures were resuspended, and OD530 readings were performed using a BioTek Epoch2 plate reader (Agilent). The mean and the standard deviation of all 24 h no-drug control OD530 readings were calculated per isolate from all plates. The EUCAST antifungal MIC methods for yeast defines the MIC for azoles and echinocandins as the lowest drug concentration that inhibits ≥50% of growth relative to no-drug control and the MIC for amphotericin B as the lowest concentration that inhibits ≥90% of growth relative to no-drug control (27). MICs for isolates with a no-drug control OD530 of >0.2 were determined according to EUCAST guidelines and interpreted according to available EUCAST breakpoint values. Per EUCAST guidelines, isolates with an OD530 ≤ 0.2 were re-incubated per EUCAST guidelines and re-read at 48 h. Isolates with an OD530 ≤ 0.2 at 48 h were re-tested by gradient diffusion strip. Fluconazole MIC screening was performed up to a maximum concentration of 32 mg/L, which exceeds clinical breakpoints. *C. glabrata*, *C. tropicalis*, and *C. utilis* isolates with fluconazole MIC values > 32 mg/L were subsequently tested at higher concentrations to determine MIC values. *C. krusei* isolates were not tested at fluconazole concentrations above 32 mg/L. Quality control for each MIC batch was performed using *C. lusitaniae* FDA-CDC AR Bank #0398 and/or *C. krusei* FDA-CDC AR Bank #0397 (60). All MIC assays were performed in triplicate.

### MIC by gradient diffusion strip

MIC testing by gradient diffusion strip was performed for isolates with no-drug control OD values ≤ 0.2 at 24 and 48 h. Antifungal susceptibility testing was adapted from the CLSI supplement M60 document protocol for gradient diffusion strips (61). Briefly, isolates were struck from glycerol stocks onto Sabouraud dextrose agar plates and incubated for 24 h at 35°C. For each culture, multiple colonies were picked, suspended in sterile water, and diluted to a final OD600 of 0.01 using a spectrophotometer. Next, 100 µL of the diluted cells was plated onto RPMI plates; a gradient diffusion strip (fluconazole: Biomerieux, product no. 510858, amphotericin B: Liofilchem, product no. 921531) was applied; and the plate was incubated for 24 h at 35°C in a humidified chamber. At 24 h, plates were imaged using a Bio-Rad Gel Doc system. The MIC values were determined by identifying the concentration where the lawn of growth intersected with the gradient strips.

### Supra-MIC growth by broth microdilution

SMG was calculated for all isolates with no-drug control OD values > 0.2 at 24 h. Plates were incubated for an additional 24 h at 35°C and resuspended. OD530 readings were performed using a BioTek Epoch2 plate reader (Agilent). SMG in fluconazole was calculated as the mean of 48 h growth in all wells above the MIC concentration, divided by the mean of the no-drug control wells. All SMG assays were performed in triplicate.

### Growth curve analysis

Overnight cultures were grown in a shaking incubator at 30°C in liquid YPAD medium with 2% dextrose (10 g/L yeast extract, 20 g/L Bacto peptone, 20 g/L dextrose, 0.04 g/L adenine, and 0.08 g/L uridine). Overnight cultures were diluted in fresh YPAD medium to a final OD600 of 0.01, and 20 µL of this cell suspension was inoculated into a 96-well plate containing 180 µL of YPAD with 1% dextrose. Cells were grown in a BioTek Epoch2 plate reader at 30°C for 24 h with constant shaking, and OD600 readings were taken every 15 min. Growth curves were performed in triplicate. Growth curve metrics, including mean and standard deviation for carrying capacity, growth rate, doubling time, AUC-E, and AUC-L, were calculated with the R package *Growthcurver* (v0.3.1) (62, 63). Metrics were plotted with the R package *ggplot2* (v3.5.1) (64).

### Correlation testing

Spearman’s rank correlation coefficient was calculated for MIC relative to SMG and to growth rate in YPAD. All correlation analyses and multiple test correction (using the Holm method) were performed with the R package *correlation* (v0.8.4) (65, 66).

## Data Availability

Scripts used in data analysis and figure generation are available at https://github.com/selmeckilab/2024_Candida_clinical_isolate_phenotyping.

## SUPPLEMENTAL MATERIAL

Figures S1 to S3 spectrum.00464-25-s0001.pdf Scatterplots of growth rate and MIC values for all species.

Tables S1 to S7 spectrum.00464-25-s0002.xlsx Strain tables, phenotyping data summaries, and statistics.

ASM does not own the copyrights to Supplemental Material that may be linked to, or accessed through, an article. The authors have granted ASM a non-exclusive, world-wide license to publish the Supplemental Material files. Please contact the corresponding author directly for reuse.
