# Supplementary material for: Heterogeneity of Candida bloodstream isolates in an academic medical center and affiliated hospitals
Source: Microbiol Spectr. 2025 Jun 23;13(8):e00464-25. doi: 10.1128/spectrum.00464-25 (PMC12323643; doi:10.1128/spectrum.00464-25)
Supplement: Figures S1 to S3 — Scatterplots of growth rate and MIC values for all species. [file spectrum.00464-25-s0001.pdf]

SUPPLEMENTARY INFORMATION (Scott *et al*. Heterogeneity of *Candida* bloodstream isolates in an academic medical center and affiliated hospitals)

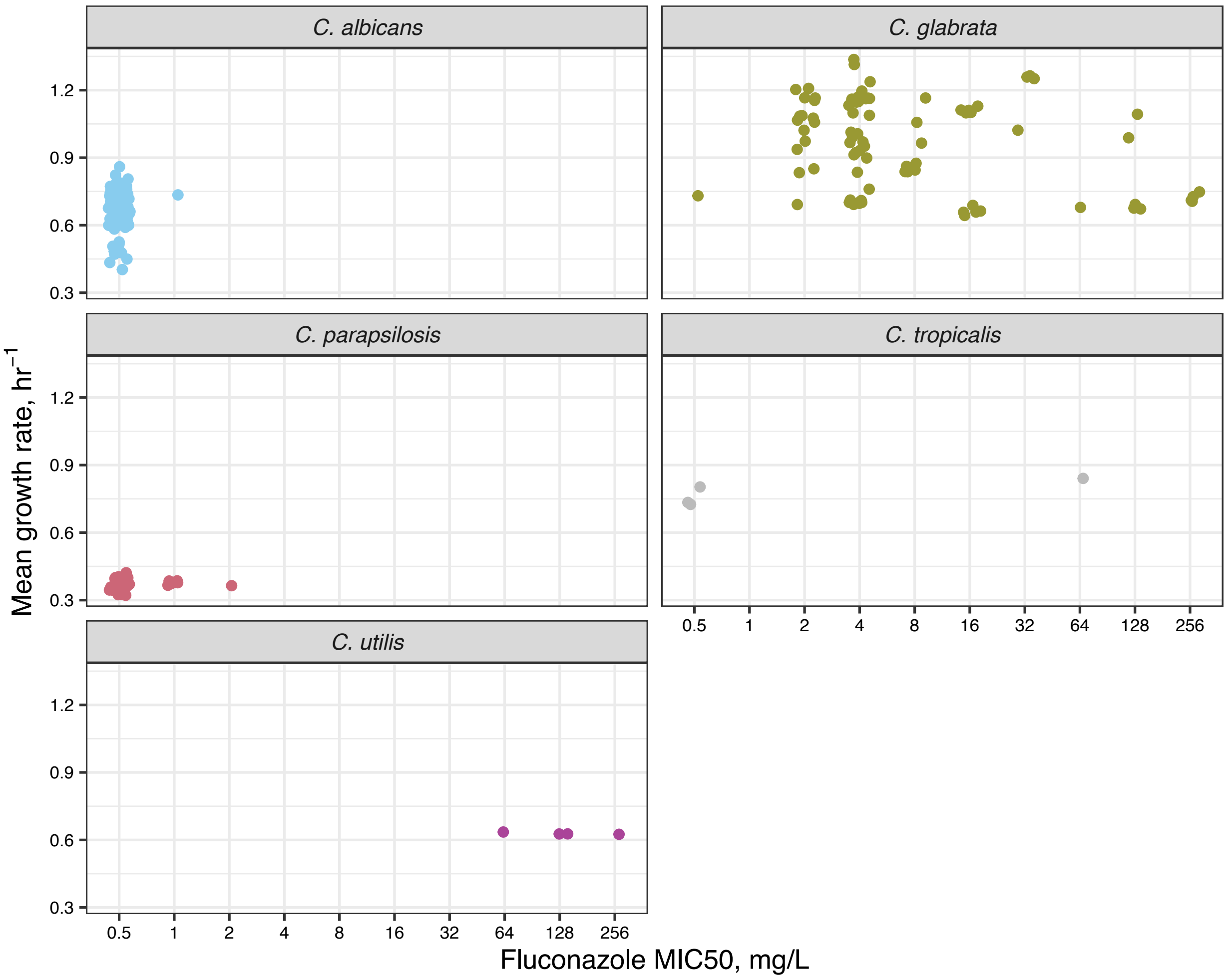

Supplementary Figure S1. Growth rate is negatively correlated with increasing fluconazole MIC values in *C. glabrata* and *C. utilis*. Scatterplot of fluconazole MIC (x-axis) and mean growth rate/hr (y-axis). There is no correlation in *C. albicans*, *C. parapsilosis* and *C. tropicalis*.

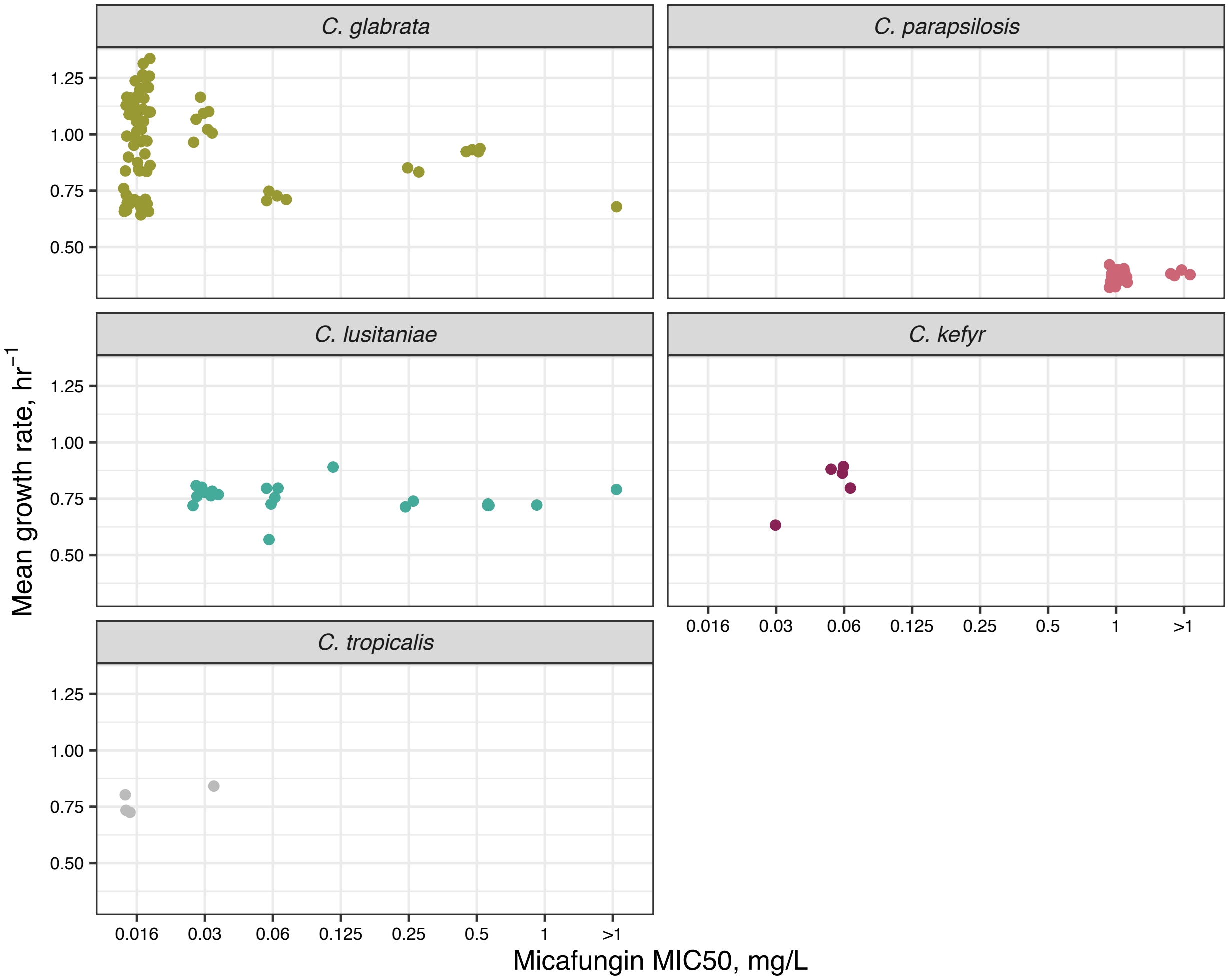

Supplementary Figure S2. Growth rate is not correlated with micafungin MIC for any species. Scatterplot of micafungin MIC (x-axis) and mean growth rate/hr (y-axis).

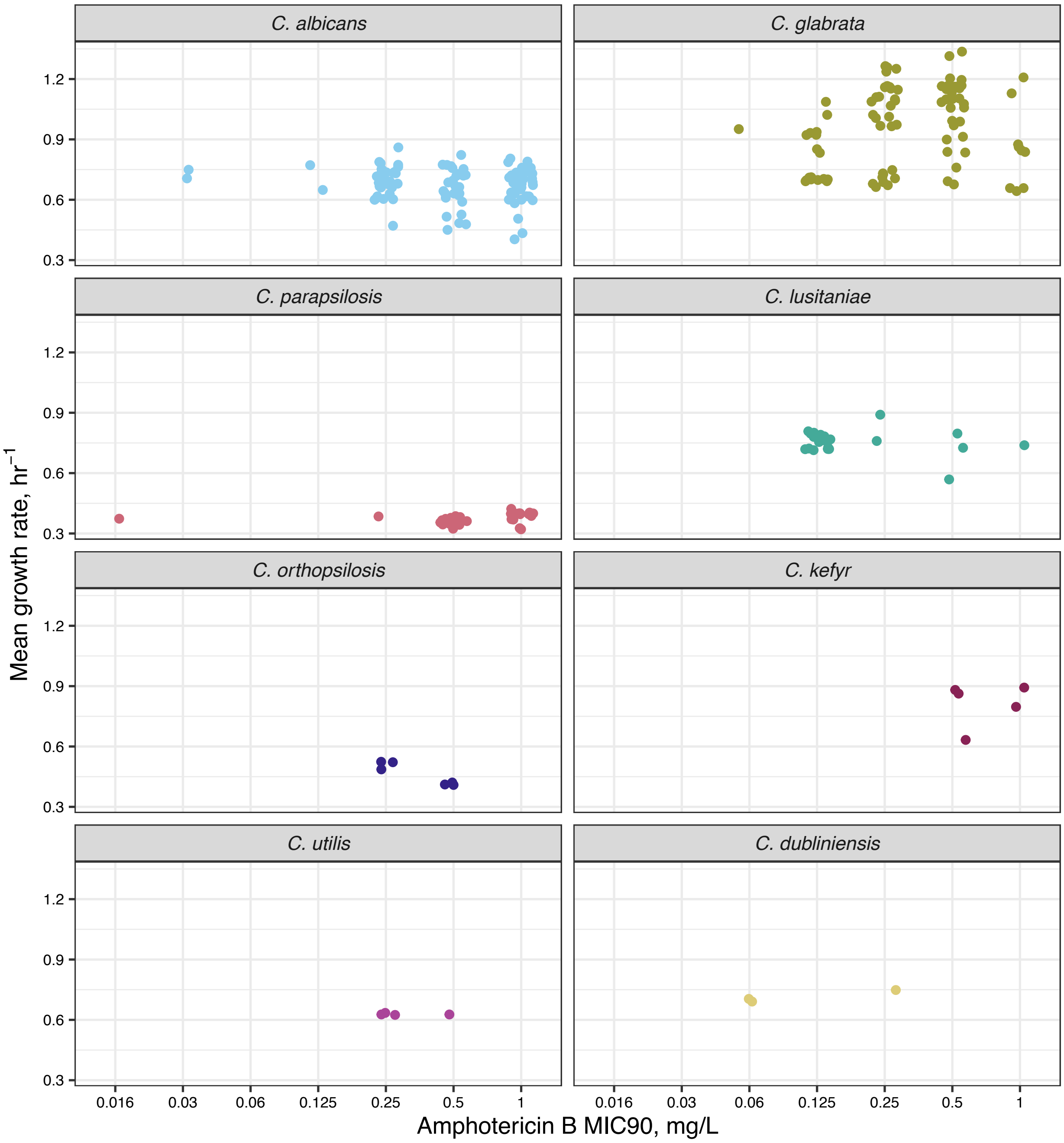

Supplementary Figure S3. Growth rate is negatively correlated with increasing amphotericin MIC values in *C. orthopsilosis* but positively correlated in *C. glabrata* and *C. parapsilosis*. Scatterplot of amphotericin B MIC (x-axis) mean growth rate/hr (y-axis). There is no correlation in *C.albicans*, *C. lusitaniae*, *C. kefyr*, *C. utilis* or *C. dubliniensis*.

Supplementary Data (excel)
Supplementary Table S1: Strain, MIC, SMG table
Supplementary Table S2: Serial isolate cases
Supplementary Table S3: Recurrent and polyfungal cases
Supplementary Table S4: Within-patient MIC differences
Supplementary Table S5: Fluconazole SMG summary statistics
Supplementary Table S6: Within-patient fluconazole SMG differences
Supplementary Table S7: Growth rate and MIC correlation for all species-drug combinations having more than 1 MIC value
